# The Impact of Web-Based Feedback on Physical Activity and Cardiovascular Health of Nurses Working in a Cardiovascular Setting: A Randomized Trial

**DOI:** 10.3389/fphys.2018.00142

**Published:** 2018-03-06

**Authors:** Jennifer L. Reed, Christie A. Cole, Madeleine C. Ziss, Heather E. Tulloch, Jennifer Brunet, Heather Sherrard, Robert D. Reid, Andrew L. Pipe

**Affiliations:** ^1^Division of Cardiac Prevention and Rehabilitation, University of Ottawa Heart Institute, Ottawa, ON, Canada; ^2^Faculty of Health Sciences, School of Human Kinetics, University of Ottawa, Ottawa, ON, Canada

**Keywords:** nurses, physical activity, cardiovascular, web-application, activity monitor, challenges

## Abstract

A disconcerting proportion of Canadian nurses are physically inactive and report poor cardiovascular health. Web-based interventions incorporating feedback and group features may represent opportune, convenient, and cost-effective methods for encouraging physical activity (PA) in order to improve the levels of PA and cardiovascular health of nurses. The purpose of this parallel-group randomized trial was to examine the impact of an intervention providing participants with feedback from an activity monitor coupled with a web-based individual, friend or team PA challenge, on the PA and cardiovascular health of nurses working in a cardiovascular setting.

**Methods:** Nurses were randomly assigned in a 1:1:1 ratio to one of the following intervention “challenge” groups: (1) individual, (2) friend or (3) team. Nurses wore a Tractivity® activity monitor throughout a baseline week and 6-week intervention. Height, body mass, body fat percentage, waist circumference, resting blood pressure (BP) and heart rate were assessed, and body mass index (BMI) was calculated, during baseline and within 1 week post-intervention. Data were analyzed using descriptive statistics and general linear model procedures for repeated measures.

**Results:** 76 nurses (97% female; age: 46 ± 11 years) participated. Weekly moderate-to-vigorous intensity PA (MVPA) changed over time (*F* = 4.022, df = 4.827, *p* = 0.002, η^2^ = 0.055), and was greater during intervention week 2 when compared to intervention week 6 (*p* = 0.011). Daily steps changed over time (*F* = 7.668, df = 3.910, *p* < 0.001, η^2^ = 0.100), and were greater during baseline and intervention weeks 1, 2, 3, and 5 when compared to intervention week 6 (*p* < 0.05). No differences in weekly MVPA or daily steps were observed between groups (*p* > 0.05). No changes in body mass, BMI or waist circumference were observed within or between groups (*p* > 0.05). Decreases in body fat percentage (−0.8 ± 4.8%, *p* = 0.015) and resting systolic BP (−2.6 ± 8.8 mmHg, *p* = 0.019) were observed within groups, but not between groups (*p* > 0.05).

**Conclusions:** A web-based intervention providing feedback and a PA challenge initially impacted the PA, body fat percentage and resting systolic BP of nurses working in a cardiovascular setting, though increases in PA were short-lived. The nature of the PA challenge did not differentially impact outcomes. Alternative innovative strategies to improve and sustain nurses' PA should be developed and their effectiveness evaluated.

## Introduction

Nurses are the largest professional group within the health care workforce. Several investigators in varied settings have assessed the self-reported and objective physical activity (PA) levels of nurses and shown low levels of PA (Kaewthummanukul et al., 2006; Sveinsdottir and Gunnarsdottir, 2008; Ratner and Sawatzky, 2009; James et al., 2013; Babiolakis et al., 2015; Perry et al., 2015; Reed et al., 2018). The *National Survey of the Work and Health of Nurses in Canada* showed that a disconcerting proportion of nurses are overweight or obese (45%) and smokers (16%); have high blood pressure (13%), high cholesterol (10%) and diabetes (3%); and, experience fair/poor mental health (6%) (Shields and Wilkins, 2006). The irrefutable evidence demonstrating the effectiveness of regular PA in the prevention and management of cardiovascular disease and associated risk factors (Warburton et al., 2006, 2010; Haskell et al., 2007; Reed and Pipe, 2016) highlights the opportunity afforded by targeted PA interventions to promote positive behavior change within this unique and large professional population.

As adults worldwide embrace modern technologies, web-based innovations may represent opportune, convenient, and cost-effective methods to target suboptimal PA levels and poor cardiovascular health of nurses. Web-based interventions that can be delivered anytime and anywhere warrant particular attention because they are accessible 24 h a day, 7 days a week which may be ideal for nurses working long (i.e., 12 h) and rotating (i.e., days, evenings, nights, weekdays, weekends) shifts. Several reviews have shown that web-based interventions can increase PA levels and reduce body mass, waist circumference and blood pressure in adults (van den Berg et al., 2007; Liu et al., 2013; Joseph et al., 2014; Seo and Niu, 2015; Direito et al., 2017; Sorgente et al., 2017).

The primary purpose of this parallel-group randomized trial was to examine the impact of a web-based intervention providing specific feedback derived from an activity monitor on the PA levels and cardiovascular health of nurses working in a Canadian cardiovascular setting. We hypothesized that nurses' PA levels and cardiovascular health would improve in response to the receipt of personalized feedback regarding their PA levels derived from an activity monitor. Further, as modern informatics capabilities enable us to harness strategies designed to initiate and support positive behavior change, the secondary purpose of this trial was to assess whether nurses' PA levels are enhanced when they work together to meet their PA goals (i.e., friend or team PA challenge) compared to when they work alone to meet their goals (i.e., individual PA challenge). We hypothesized that nurses' assigned to a friend or team PA challenge (and thus have their weekly PA levels displayed to others) would become more physically active when compared to nurses assigned to an individual PA challenge. This hypothesis is based on work suggesting that people perform better when they are in front of others than when they perform alone (Hausenblas et al., 2014). One explanation for this is based on the self-presentation theory (Leary, 1992) which suggests that the desire to enhance oneself and make positive impressions in front of others is an important motivator of human behavior. From this perspective, one might expect that nurses will increase their PA levels more if they know that others will see their levels than if no one else will see their PA levels.

## Materials and methods

### Study design

This parallel-group randomized trial was conducted at the University of Ottawa Heart Institute (UOHI), a tertiary care cardiovascular institute. This study was carried out in accordance with the consolidated standards of reporting trials (CONSORT) and intervention description and replication (TIDieR) checklists (Hoffmann et al., 2014; Boutron et al., 2017). All participants provided written informed consent in accordance with the Declaration of Helsinki. The protocol was approved by the UOHI Human Ethics Board (Protocol No. 20130429).

### Protocol

#### Recruitment

A convenience sample of participants was recruited between September and November 2013. Research staff informed nurses, administrative staff and nursing-leaders of the study by attending nursing meetings and morning rounds, and by distributing recruitment posters throughout the hospital (e.g., nursing lounges and stations, information boards, cafeterias). The posters contained a brief description of the study and contact information for the research staff. Hospital administrative staff and nursing-leaders assisted in distributing recruitment materials. Nurses interested in participating in the study contacted the research staff; screening was performed on-site.

Eligible participants were: (1) registered nurses; (2) able to walk unassisted; (3) willing to wear a stretchable ankle band which contained a PA monitoring device (i.e., accelerometer) and had access to the internet; and, (4) able and willing to provide written informed consent. Participants who: (1) were pregnant or lactating; (2) were unable to read and understand English; (3) had medical contraindications to exercise; and/or, (4) were already using an activity monitor to track their PA levels were not eligible.

#### Randomization and intervention groups

Motivation is a principal factor prompting changes in health behaviors (Teixeira et al., 2012). Framing behavior change interventions as games or competitive endeavors may be an effective strategy to motivate change (Baranowski et al., 2008). Given previous studies have shown that employing game design features in non-game contexts is effective in improving health and well-being (Johnson et al., 2016) and that the presence of others can increase performance, participants were randomly assigned in a 1:1:1 ratio to one of the following intervention groups: (1) individual, (2) friend or (3) team challenge, which allowed us to examine if the groups facilitated or inhibited behavior change. Research staff randomly allocated participants to intervention groups using the “RAND” function of a software spreadsheet program (Excel, Microsoft, Washington, USA), and notified them of their group assignment via email. Participants were provided with a: (1) unique username and password to access the online Tractivity® program which contained their individual, friend or team challenge; and, (2) Bluetooth USB key which enabled them to upload their activity monitor data into the online Tractivity® program.

Participants could monitor their distance (km), steps (number), active time (minutes) and calories (kcal) expended on an hourly, daily, weekly, and monthly basis in a graphical format in the online Tractivity® program (see Supplementary Figure 1). In the friend and team challenge groups, group features were added such that participants' PA levels were displayed to others in their group as a means to enhance motivation to perform well. Specifically, participants randomized to the friend challenge could also monitor the total distance (km) and steps (number) of another participant randomized to the friend challenge in a graphical format in the online Tractivity® program (see Supplementary Figure 2). Participants randomized to the team challenge could also monitor the total distance (km) and steps (number) of their team and other teams in a graphical format in the online Tractivity® program (see Supplementary Figure 3). For the team challenge, five groups of five participants were created, totaling 25 participants. Participants were blinded such that no-one knew the identity of the other person or persons in their group (to comply with ethical codes of conduct).

#### Study assessments

##### Physical activity

Participants wore a Tractivity® activity monitor (Tractivity®, Vancouver, BC) held in a stretchable ankle band during waking hours throughout a baseline week and 6-week intervention, excluding periods when they engaged in water-related activities (e.g., bathing, swimming). The Tractivity® activity monitor is a lightweight, compact accelerometer that uses a proprietary signal processing algorithm to determine step counts in 1-min intervals. The activity monitor provides no visible feedback on the device and stores up to 30 days of data (i.e., distance, steps, active time, calories).

Research staff uploaded the participants' activity data into the online Tractivity® program at the end of the baseline week and 6-week intervention. Participants uploaded their activity data at times and frequencies of their choosing throughout the 6-week intervention. The Tractivity® activity monitor has been shown to be a valid measure of step counts in comparison to direct observation with less than a 0.5% error across a range of walking speeds (2.4, 3.1, 3.5, and 4.1 mph) (Warburton et al., 2013). Activity monitors were calibrated for stride length prior to the baseline week by having nurses walk 10 steps (at their usual walking speed) in a straight line on a large indoor track. These measures were performed in triplicate, and the average was entered into the online Tractivity® program to assist the proprietary signal processing algorithm in calculating step counts.

Tractivity® provided us with consecutively ordered minute-by-minute activity monitor data [i.e., steps, distance (km), active time (minutes), calories (kcal)] for each day of the baseline and intervention phases for all participants. We used a Hypertext Preprocessor (PHP, version 7.0) script to process the data. All activity monitor data were screened to identify valid and non-valid days. Only days with at least 10 h of wear-time were retained for analyses, as a minimum accelerometer wear-time of 10 h has been used to provide a valid measure of daily PA (Troiano et al., 2008). Activity monitor determined step counts were used to calculate steps, minutes of MVPA and number of days PA guidelines of ≥150 min/week of MVPA in bouts of ≥10 min were met (Canadian Society for Exercise Physiology, 2011; World Health Organization, 2011). Using published guidelines (Tudor-Locke et al., 2005), a threshold value of at least 100 steps/minute was used to define MVPA. Weekly MVPA minutes in bouts of ≥10 min and daily steps were calculated.

##### Cardiovascular health indicators

Cardiovascular health measures were taken between 0630 and 1000 hours. Height was measured to the nearest 0.1 cm, body mass was measured to the nearest 0.1 kg, and body mass index (BMI) was calculated (kg/m^2^). Waist circumference was measured to the nearest 0.5 cm (Seca 201) at the narrowest point of the torso while participants stood with arms at their sides, feet together and abdomen relaxed (American College of Sports Medicine, 2017). Body fat percentage was measured using bioelectrical impedance (BIA) (UM-041, Tanita, Roxton Industries Inc., Kitchener, Ontario). Participants were asked to adhere to the following prior to their anthropometric measurements: (1) no eating or drinking for 4 h; (2) no MVPA for 12 h; (3) no alcohol consumption for 48 h; (4) to void their bladder (within 30 min); (5) to refrain from consuming caffeine and diuretic use unless prescribed by a physician; and, (6) to postpone measurement if retaining water due to changes in menstrual cyclicity. Resting blood pressure (BP) and heart rate were assessed in a seated position after a 5-min rest period using an automated, non-invasive BP monitor (Bp-TRU, Coquitlam, BC, Canada). All measures were performed in triplicate at baseline and within 1 week post-intervention, and the average was reported for descriptive purposes. Research staff collecting cardiovascular health measures were blinded to participants' group assignment.

### Statistical analyses

Analyses were performed using SPSS for Windows (version 24; IBM Corp, Armonk, NY, USA). A complete case analysis was performed; only 4% (*n* = 3/75) of the randomized participants did not complete the intervention. All outcome variables were tested for normality using Shapiro-Wilk tests of normality; number of days activity monitors were worn, MVPA levels, steps, and cardiovascular health indicators [body mass, BMI, waist circumference, resting BP (baseline phase)] were not normally distributed.

Friedman's two-way analysis of variance (ANOVA) by ranks summary was performed to examine changes in the number of days the activity monitors were worn throughout the baseline and intervention phases. A two-step approach for transforming continuous non-normalized variables to normal was applied to the MVPA levels and steps variables (Templeton, 2011). A one-way ANOVA was performed to examine differences in the normalized weekly MVPA levels and daily steps between intervention groups at baseline. A two-way repeated measures ANOVA was performed to examine changes in the normalized MVPA levels and steps variables throughout the baseline and intervention phases both within and between groups (i.e., individual, friend and team); significant values were adjusted using Bonferroni correction for multiple tests. Wilcoxon signed rank tests were performed to compare cardiovascular health indicators between time points (i.e., baseline and within 1 week post-intervention), and Kruskal Wallis tests were performed to compare changes (i.e., post-intervention values—baseline values) in cardiovascular health indicators between groups. Non-normalized values are presented in the results for descriptive purposes. Data are reported as means ± standard deviations, unless otherwise noted, and *p* < 0.05 was considered statistically significant. Our *post-hoc* power analysis revealed that an eta-squared value of 0.022 (i.e., small effect size) and alpha of 0.05, a sample size of 76 participants provides adequate power (1–β = 0.92) to detect significant differences in PA within (i.e., baseline and intervention weeks 1–6) and between groups.

## Results

### Participants

**Figure 1:**
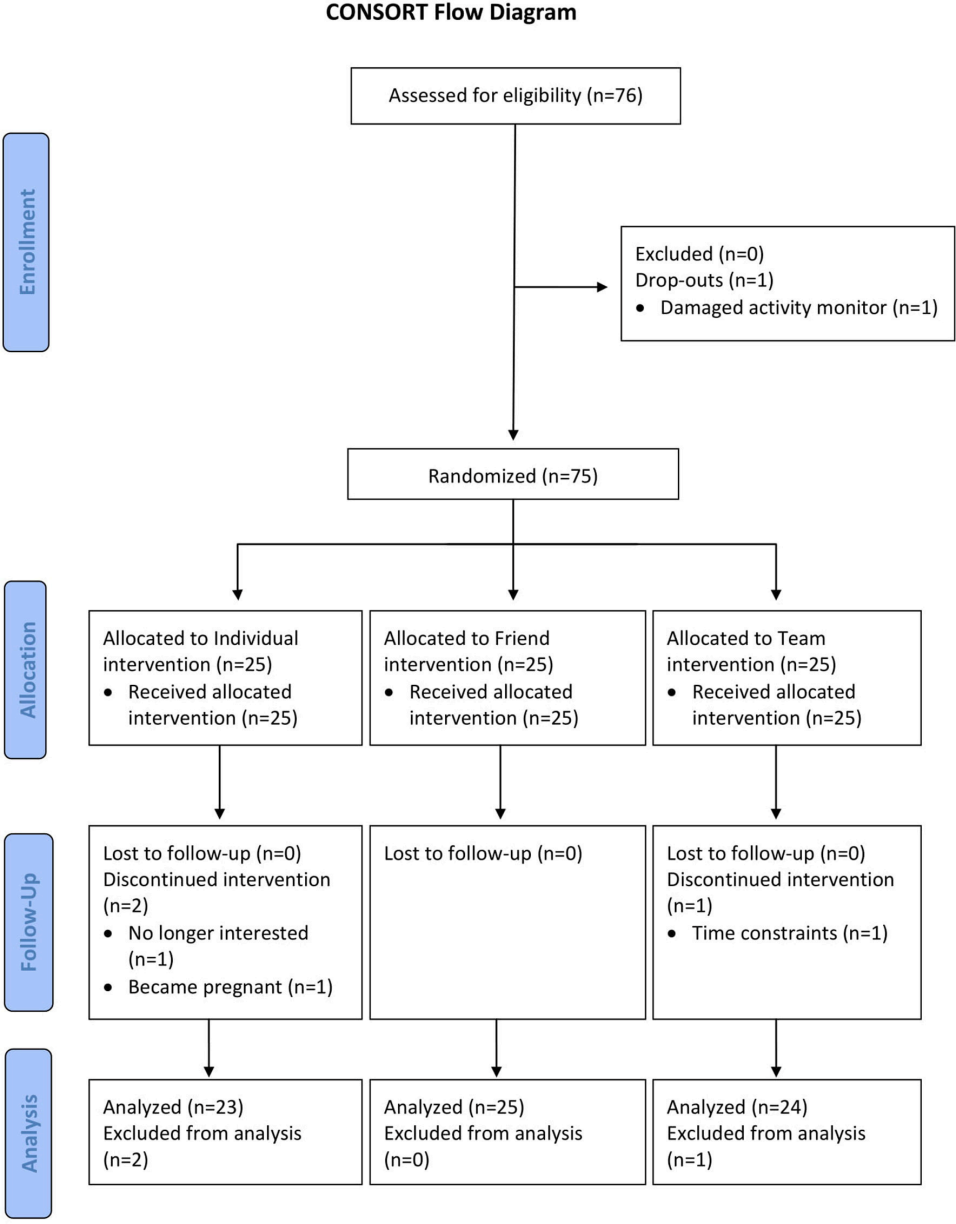
CONSORT flow diagram of nurses recruited and reasons for withdrawals. CONSORT, Consolidated Standards of Reporting Trials; PA, physical activity.

All 76 screened participants met study eligibility criteria and consented to participate; 75 were randomized to the individual, friend and team PA challenges (see Figure 1).

Nurses' demographics, anthropometrics, types of work shifts, and nursing roles are presented in Table 1. On average, nurses were categorized as being overweight, normotensive, with a low-risk waist circumference according to the American College of Sports Medicine (ACSM) guidelines (American College of Sports Medicine, 2017). Most were female (97%), working days (53%), and performing clinical duties (72%). They spent an average of 27.4±49.1 min/week in MVPA in bouts of ≥10 min; only three (4%) nurses met current PA guidelines at baseline.

**Table 1:** Participant characteristics.

**Demographics and anthropometrics**	**All participants (*N* = 76) Mean ± SD**	**Females (*n* = 74) Mean ± SD**	**Males (*n* = 2) Mean ± SD**
Age (years)	46.3 ± 10.9	46.6 ± 10.8	36.0 ± 15.6
Sex (% female)	97%	100%	0%
Height (cm)	165.4 ± 6.8	165.0 ± 6.1	179.3 ± 19.4
Body mass (kg)	75.4 ± 16.4	75.4 ± 16.4	73.0 ± 25.5
BMI (kg/m^2^)	27.5 ± 5.6	27.7 ± 5.6	22.2 ± 3.1
Waist circumference (cm)	84.1 ± 12.6	84.2 ± 12.6	83.7 ± 17.5
Resting systolic blood pressure (mmHg)	115 ± 12	115 ± 13	116 ± 6
Resting diastolic blood pressure (mmHg)	75 ± 8	75 ± 8	82 ± 4
Resting heart rate (bpm)	68 ± 9	68 ± 9	69 ± 9
Weekly hours	36.3 ± 8.3	36.2 ± 8.4	42.8 ± 7.4
Types of shifts^*^	**n (%)**		
Working days only	40 (52.6)	40 (54.1)	–
Working days and nights	29 (38.2)	27 (36.5)	2 (100.0)
Working nights only	5 (6.6)	5 (6.8)	–
Nursing roles^*^	**n (%)**		
Clinical only	53 (71.6)	51 (70.8)	2 (100.0)
Clinical and managerial	6 (8.1)	3 (4.2)	–
Clinical and research	3 (4.1)	3 (4.2)	–
Managerial only	9 (12.2)	9 (12.5)	–
Research only	3 (4.1)	6 (8.3)	–

*BMI, body mass index; SD, standard deviation*.

* *, missing n = 2 for females for types of shifts and nursing roles*.

### Dropouts

One participant dropped out after baseline due to a damaged device, and three (4%) participants dropped out during the intervention due to pregnancy (*n* = 1), loss of interest (*n* = 1), or time constraints (*n* = 1). Overall, 72 (96%) of the randomized participants completed all study assessments, including 23 (92%) assigned to the individual challenge, 25 (100%) assigned to the friend challenge, and 24 (96%) assigned to the team challenge.

### Adherence to intervention

Nurses wore the activity monitor for at least 10 h/day for an average of 31 of the total 42 intervention days (overall compliance rate of 74%). The number of days the nurses wore the activity monitor decreased significantly throughout the baseline and intervention phases (*p* < 0.05). Nurses wore the activity monitor for ≥10 h/day for an average of 6.0 ± 1.9 (baseline), 6.0 ± 2.0 (intervention week 1), 5.8 ± 1.8 (intervention week 2), 5.9 ± 1.9 (intervention week 3), 4.6 ± 2.0 (intervention week 4), 4.8 ± 2.4 (intervention week 5), and 3.5 ± 3.0 (intervention week 6) days. No significant differences in the number of days the nurses wore the activity monitor were observed between intervention groups (*p* > 0.05).

### Effects of intervention on physical activity

**Figure 2:**
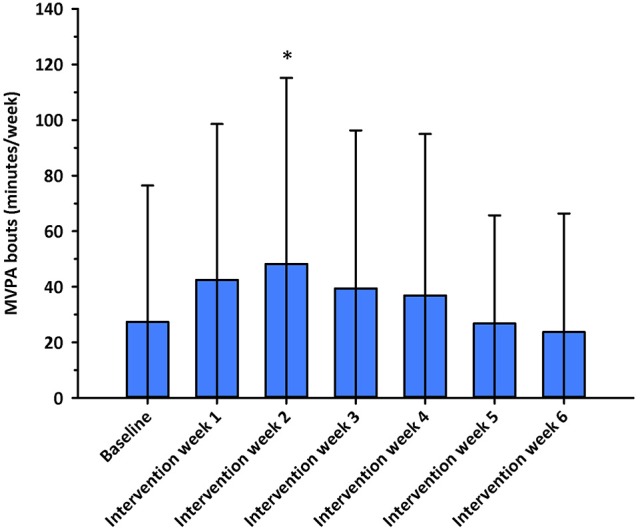
Minutes/week in moderate-to-vigorous intensity physical activity in bouts of at least 10 min across the baseline and intervention phases. ^*^p < 0.05 vs. intervention week 6. MVPA, moderate-to-vigorous intensity physical activity.

**Figure 3:**
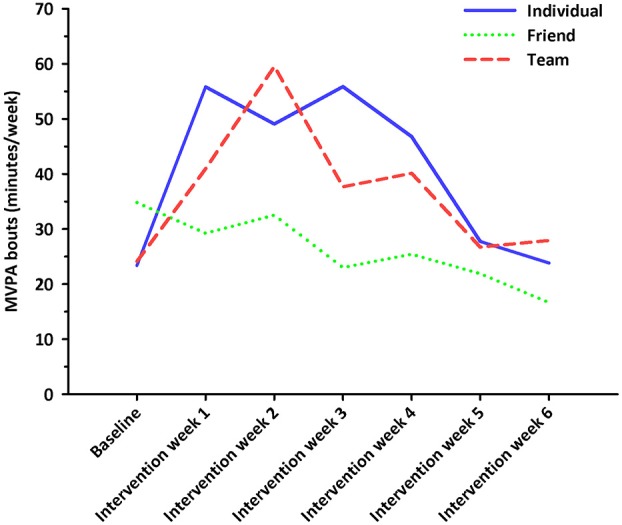
Minutes/week in moderate-to-vigorous intensity physical activity in bouts of at least 10 min across the baseline and intervention phases for participants in the individual, friend and team challenges. Solid blue line represents the individual challenge. Short dotted green line represents the friend challenge. Long dotted red line represents the team challenge.

**Figure 4:**
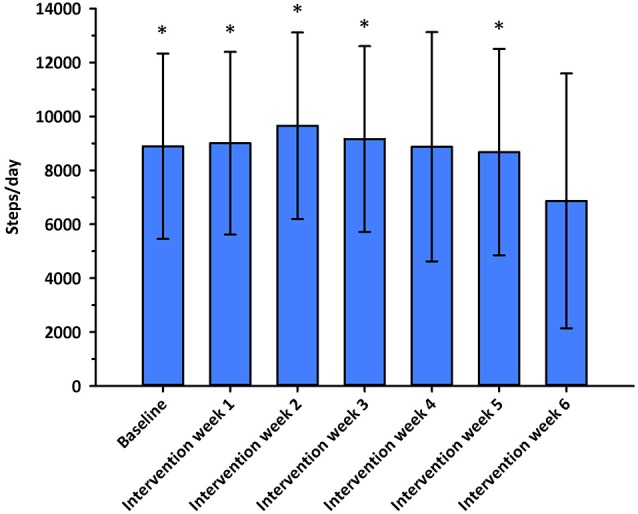
Average steps/day across the baseline and intervention phases. ^*^p < 0.05 vs. intervention week 6.

**Figure 5:**
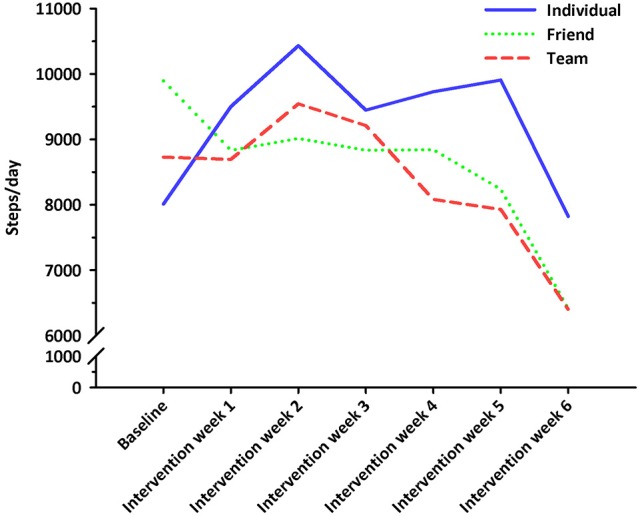
Average steps/day across the baseline and intervention phases for participants in the individual, friend and team challenges. Solid blue line represents the individual challenge. Short dotted green line represents the friend challenge. Long dotted red line represents the team challenge.

No significant differences in nurses' weekly MVPA levels (*F* = 0.407, *p* = 0.667, η^2^ = 0.01) or daily steps (*F* = 1.696, *p* = 0.191, η^2^ = 0.046) were observed between intervention groups at baseline. Nurses' weekly MVPA levels changed significantly over time (*F* = 4.022, df = 4.827, *p* = 0.002, η^2^ = 0.055), and were greater during intervention week 2 when compared to intervention week 6 (*p* < 0.05; see Figure 2). No significant differences in MVPA levels were observed between intervention groups (*F* = 1.199, df = 9.654, *p* = 0.292, η^2^ = 0.034; see Figure 3). Nurses' daily steps changed significantly over time (*F* = 7.668, df = 3.910, *p* < 0.001, η^2^ = 0.100), and were greater during baseline and intervention weeks 1, 2, 3, and 5 when compared to intervention week 6 (*p* < 0.05) (see Figure 4). No significant differences in daily steps were observed between intervention groups (*F* = 1.146, df = 7.819, *p* = 0.333, η^2^ = 0.032; see Figure 5). Two nurses (3%) nurses met current PA guidelines post-intervention.

### Effects of intervention on cardiovascular health indicators

Nurses' cardiovascular health parameters are presented in Table 2. No significant changes in body mass, BMI or waist circumference were observed between baseline and within 1 week post-intervention (*p* > 0.05). Significant decreases in body fat percentage and resting systolic BP were observed within 1 week post-intervention when compared to baseline (*p* < 0.05). No significant differences in changes in cardiovascular health indicators were observed between groups (*p* > 0.05).

**Table 2:** Participants' cardiometabolic health at baseline and post-intervention.

**Variables**	**Baseline mean ± SD**	**Post-intervention mean ± SD**	**Change mean ± SD**	**Effect size (*r*)**	***p*-values**
Body mass (kg)	75.9 ± 16.6	75.2 ± 15.9	−0.6 ± 3.4	−0.16	0.570
BMI (kg/m^2^)	27.6 ± 5.7	27.3 ± 5.3	−0.3 ± 1.2	−0.21	0.460
Body fat (%)	36.7 ± 8.7	35.9 ± 8.4	−0.8 ± 4.8	−0.70	0.015
Waist circumference (cm)	84.0 ± 12.7	83.6 ± 12.5	−0.4 ± 1.7	−0.54	0.063
Resting SBP (mmHg)	114.6 ± 12.4	111.9 ± 11.3	−2.6 ± 8.8	−0.68	0.019

*BMI, body mass index; SBP, systolic blood pressure; SD, standard deviation*.

## Discussion

This is the first randomized trial, to our knowledge, to examine the impact of a web-based intervention incorporating feedback and group features on the PA levels and cardiovascular health of nurses working in a cardiovascular setting. We observed initial increases in nurses' PA levels (i.e., weekly MVPA levels and daily steps), though these were not sustained over the 6-week intervention and few met the current PA guidelines (≥150 min/week in bouts of ≥10 min). We also observed improvements in nurses' body fat percentage and resting systolic BP. Introducing web-based group features (i.e., friend and team PA targets) for motivation did not differentially impact PA or cardiovascular outcomes.

Nurses reach a large proportion of the population making them a critically important element of the health-care workforce. Nursing practice is physically and psychologically demanding (Chin et al., 2016). Physical inactivity and cardiovascular disease have been shown to be related to lower ability to work and a greater incidence of absenteeism (Burton et al., 2014; van den Berg et al., 2017). Improving the lifestyle and overall well-being of nurses is important in order to permit optimal patient care. E-health broadly refers to the use of emerging information and communication technology to improve or enable health and health care (Government of Canada, 2010). E-health encompasses a wide range of services or systems, including electronic health records, e-prescribing, telemedicine (e.g., online and telephone coaching), consumer health informatics (e.g., on demand educational content), wearable devices (e.g., Tractivity™, Fitbit™) and real-time monitoring of user health and behavioral data. Evidence has suggested that e-health interventions may improve the PA levels and health outcomes of adults (Beratarrechea et al., 2014; Joseph et al., 2014; Direito et al., 2017). Providing web-based feedback from wearable devices is acceptable and can increase the PA levels of inactive overweight and obese women (Cadmus-Bertram et al., 2015a,b). Our work extends these findings to nurses and provides support for the use of e-health interventions to target PA and cardiovascular outcomes in nurses working in a cardiovascular setting as revealed by the good compliance with the intervention and initial effects on behavior and health outcomes.

We found that web-based feedback from an activity monitor resulted in immediate increases in nurses' PA levels (i.e., weekly MVPA levels and daily steps). Yet, consistent with other web-based PA interventions demonstrating short-lived increases in PA levels (Vandelanotte et al., 2007; Kernot et al., 2014; Fjeldsoe et al., 2015; Joseph et al., 2015), nurses' PA levels decreased mid-way through the current intervention. Our 6-week intervention was relatively short in duration and incorporated a limited range of motivational strategies, namely group features. It could be argued that a longer intervention that combines feedback with additional strategies (e.g., social support, autonomy support, offering value-based rationales for PA) within the web platform is needed to achieve and sustain improvements in PA levels, particularly for those who are not meeting current PA guidelines. Whether web-based interventions that encompass more strategies that can intrinsically motivate PA are more effective in sustaining nurses' PA levels requires further investigation.

We attempted to assist nurses in achieving PA targets by having participants share data with other nurses to motivate the initiation and maintenance of PA; this appeared to lack sustained appeal. We chose friend and team PA challenges based on research showing that: (1) the act of self-monitoring can improve PA levels (Michie et al., 2009); (2) social factors (e.g., social learning, comparison, normative influence, facilitation, cooperation, recognition) can be a powerful tool for increasing the effectiveness of web-based interventions (Matthews et al., 2016); (3) social competition via the web can motivate participants to become more physically active when compared to self-monitoring only (Prestwich et al., 2017); (4) gamification can have a positive impact on behavioral and health outcomes (Johnson et al., 2016); and, (5) the desire to enhance oneself and make positive impressions is an important motivator of human behavior (Leary, 1992). The integration of technological-mediated group features did not, however, impact nurses' PA levels or cardiovascular health when compared to those who did not have access to these features. One explanation for the null finding is that the anonymity of nurses within the groups prevented social support and relatedness between participants. It is possible that nurses would have accumulated greater PA levels if the friend and team challenge conditions allowed them to feel connected and accountable to their friend or team members. It is also possible that this type of group feature is insufficient to change PA unless cash or prize incentives are provided. Finally, feedback may have undermined nurses' intrinsic motivation to perform PA because providing social pressures and rewards can give rise to extrinsic motivation (Deci and Ryan, 2012; DeSmet et al., 2014). Strategies which foster intrinsic motivation (e.g., when the behavior is done for enjoyment and personal satisfaction) may better promote behavior change and maintenance (Teixeira et al., 2012; Hancox et al., 2017; Quested et al., 2017). Future research developing and testing strategies to motivate nurses and engage them in PA is warranted.

We observed statistically significant improvements in body fat percentage (−0.8%), yet no changes in body mass, BMI or waist circumference. These latter findings were not surprising given that increases in nurses' PA levels were short-lived over the 6-week intervention and likely produced minimal, if any, deficits in energy expenditure (Reed et al., 2013). We also observed a significant improvement in resting systolic BP (−2.6 mmHg). Strong evidence from a meta-analysis of randomized controlled trials of exercise training in healthy adults (5,223 participants: 3,401 exercise training participants and 1,822 sedentary controls) suggests that resting systolic BP is reduced (−3.5 mmHg) after endurance exercise (Cornelissen and Smart, 2013). The decrease (−2.6 mmHg) we observed may not be clinically significant, yet the direction of change is nevertheless favorable and associated with reduced cardiovascular morbidity and mortality (Hansson, 1996; Padwal et al., 2016). Our findings contrast those of a pedometer-based PA program for nurses in a Canadian multi-site health care center which reported no changes in resting systolic BP (Lavoie-Tremblay et al., 2014). This study, however, used self-reported BP measures which have been shown to have moderate agreement with measured BP (Taylor et al., 2010) and did not observe significant increases in PA levels.

Our study has several strengths. It is the first to examine the impact of web-based feedback from an activity monitor on the PA levels and cardiovascular health of nurses working long and rotating shifts in a cardiovascular health center. This is particularly important as innovative interventions are needed to address at-risk nursing populations (Reed et al., 2018). Second, we integrated technologically-mediated social participation into the web-based intervention to increase participants' motivation, although this did not impact PA levels or cardiovascular health indicators. Third, nurses' PA levels were objectively measured in 1-min increments throughout a baseline week and 6-week intervention using a valid activity monitor. Fourth, we observed a low dropout rate of 5% (*n* = 4/76). A review of internet- and web-based PA interventions in which the majority of participants were women revealed a dropout rate of 21% for interventions <6 months in duration (Joseph et al., 2014). Further, a pedometer-based PA program for nurses in a Canadian multi-site health care center reported a response rate of only 55% (Lavoie-Tremblay et al., 2014).

Several limitations warrant discussion. First, the generalizability of our findings to male nurses is limited as 97% of our sample were female—characteristic of the Canadian nursing population (Shields and Wilkins, 2006). Second, the generalizability of our findings to older nurses and those working nights only is limited given most of our nurses were middle age and working days only. Third, this was a single-center study. Replication of this study across several hospitals is needed to confirm our findings. Fourth, we recruited 19% of nurses from the hospital (total nursing population = approximately 400 nurses); it is possible that nurses interested in participating in a PA and health study may be “healthier” and more active than average, thus limiting the impact of a PA intervention to improve PA and cardiovascular health. Finally, we cannot affirm that participants did not disclose their group assignment to one another, and consequently contaminate the group effects. No differences in PA or cardiovascular outcomes were, however, observed between groups.

## Conclusions

Web-based PA interventions may be effective in initiating, but not sustaining optimal PA levels among Canadian nurses working in a cardiovascular setting. Improvements in nurses' body fat percentage and resting systolic BP were observed following the intervention. Embedding technologically-mediated social participation did not appear to impact nurses' PA levels or cardiovascular health. Nurses working in a cardiovascular setting do not appear to be meeting PA guidelines. Future larger multi-site randomized controlled trials are needed to confirm our findings. If our findings are replicated, alternative novel and multi-faceted interventions are needed to address the low PA levels and poor cardiometabolic health of at-risk Canadian nurses.

The growth in e-health interventions is occurring rapidly. It is foreseeable that new technologies (e.g., global positioning systems, smart watches, video games) will provide additional means of improving and or maintaining PA which is a known modifiable risk factor of cardiometabolic health. Consumers will be able to monitor their time spent in MVPA, daily steps and bouts of sedentary time. The cost of such technologies will, in all likelihood, continue to decrease as companies strive to provide competitive, accessible and affordable products for consumers. Future work is needed to synthesize all available data regarding the effectiveness of e-health interventions in improving PA levels and cardiometabolic health in adults, particularly in women as over half of women lack knowledge of cardiovascular disease risk factors and the majority are uninformed when it comes to their own level of risk (McDonnell et al., 2014; Reed et al., 2015).

## Author contributions

JR: Conceptualized and designed the study; JR: Performed the analyses and interpretations of the data, drafted the initial manuscript, and revised and approved the final manuscript as submitted; CC and MZ: Assisted with the acquisition of data, drafting and revising the manuscript; JB: Assisted in drafting and revising the manuscript; HT, HS, RR, and AP: Assisted in designing the study, selecting outcome measures, and provided critical revision of the manuscript.

### Conflict of interest statement

The authors declare that the research was conducted in the absence of any commercial or financial relationships that could be construed as a potential conflict of interest.

## Supplementary material

Supplementary Figure 1 Online Tractivity® program which displayed participants distance, steps, active time and calories expended on an hourly, daily, weekly, and monthly basis.

Supplementary Figure 2 Friend challenge in online Tractivity® program which displayed the total distance and steps of another participant randomized to the friend challenge.

Supplementary Figure 3 Team challenge in online Tractivity® program which displayed the total distance and steps of others teams randomized to the team challenge.
