# Supplementary figures and images for: The Impact of Web-Based Feedback on Physical Activity and Cardiovascular Health of Nurses Working in a Cardiovascular Setting: A Randomized Trial
Source: Front Physiol. 2018 Mar 6;9:142. doi: 10.3389/fphys.2018.00142 (PMC5845721; doi:10.3389/fphys.2018.00142)

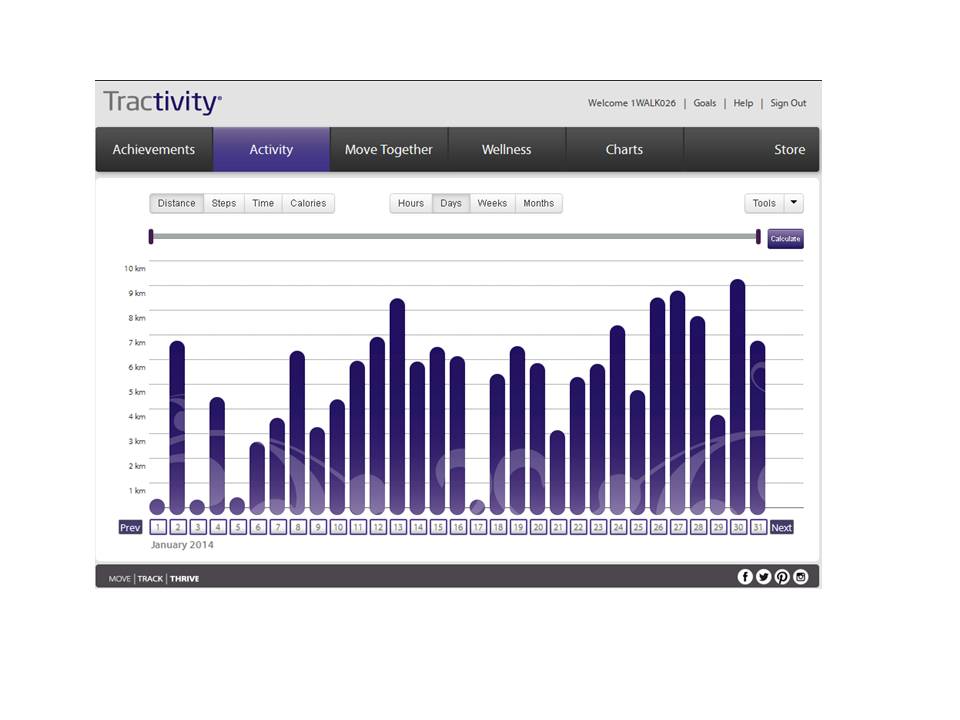

Supplement: Supplementary Figure 1 — Online Tractivity® program which displayed participants distance, steps, active time and calories expended on an hourly, daily, weekly, and monthly basis. [file Image1.JPEG]

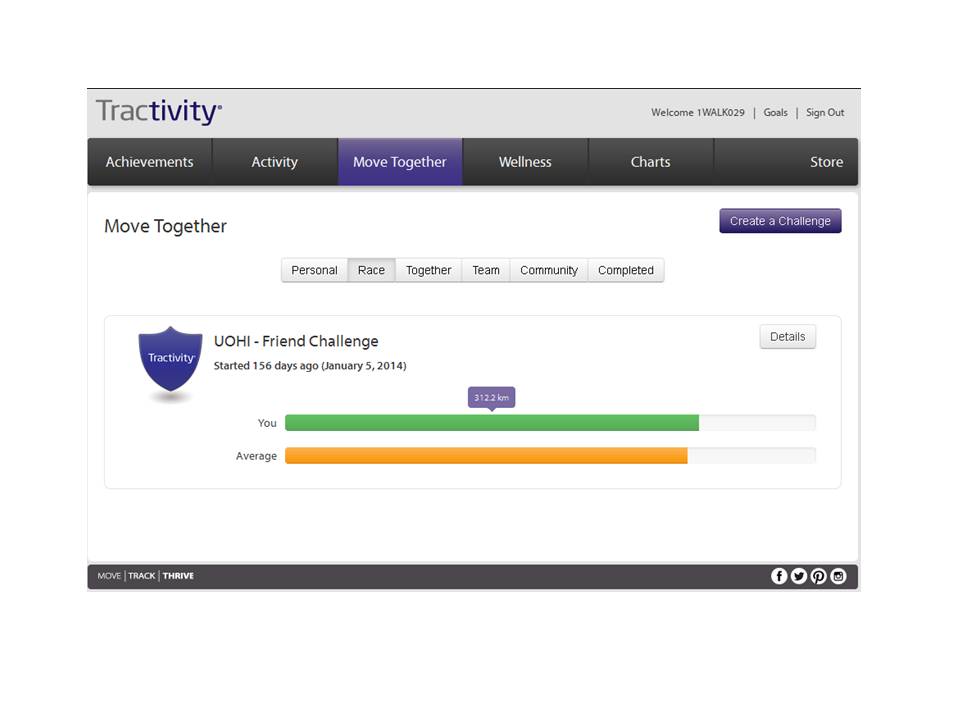

Supplement: Supplementary Figure 2 — Friend challenge in online Tractivity® program which displayed the total distance and steps of another participant randomized to the friend challenge. [file Image2.JPEG]

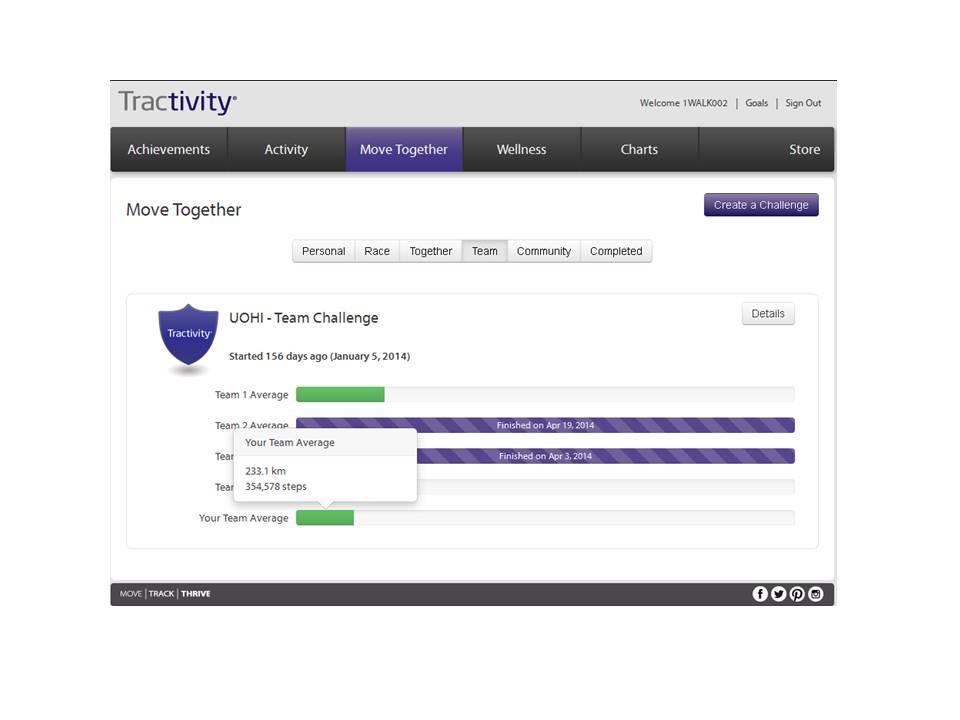

Supplement: Supplementary Figure 3 — Team challenge in online Tractivity® program which displayed the total distance and steps of others teams randomized to the team challenge. [file Image3.JPEG]
